# Single and double pain responses to individually titrated ultra-short laser stimulation in humans

**DOI:** 10.1186/s12871-019-0702-1

**Published:** 2019-03-04

**Authors:** Anna Sellgren Engskov, Agneta Troilius Rubin, Jonas Åkeson

**Affiliations:** 1 Department of Clinical Sciences Malmö, Anaesthesiology and Intensive Care Medicine, Lund University, Skåne University Hospital, Carl Bertil Laurells gata 9, 3rd floor, SE-20502 Malmö, Sweden; 2 Dermatology, Lund University, Skåne University Hospital, Malmö, Sweden

**Keywords:** Humans, Lasers, Nerve fibers, Nociceptors, Nociceptive pain, Pain measurement, Visual analog scale

## Abstract

**Background:**

This preclinical study in humans was designed to selectively induce delayed nociceptive pain responses to individually titrated laser stimulation, enabling separate bedside intensity scoring of both immediate and delayed pain.

**Methods:**

Forty-four (fourteen female) healthy volunteers were subjected to repeated nociceptive dermal stimulation in the plantar arc, based on ultra-short carbon dioxide laser with individually titrated energy levels associated with mild pain.

**Results:**

Data was analysed in 42 (12 female) subjects, and 29 of them (11 females) consistently reported immediate and delayed pain responses at second-long intervals to each nociceptive stimulus. All single pain responses were delayed and associated with lower levels (*p* = 0.003) of laser energy density (median 61; IQR 54–71 mJ/mm^2^), compared with double pain responses (88; 64–110 mJ/mm^2^). Pain intensity levels associated with either kind of response were readily assessable at bedside.

**Conclusions:**

This study is the first one to show in humans that individually titrated ultra-short pulses of laser stimulation, enabling separate pain intensity scoring of immediate and delayed responses at bedside, can be used to selectively induce and evaluate delayed nociceptive pain, most likely reflecting C-fibre-mediated transmission. These findings might facilitate future research on perception and management of C-fibre-mediated pain in humans.

## Background

Nociceptive pain in man, peripherally transmitted by myelinized Aδ- and non-myelinized C-fibres, is characterized by immediate, mainly pricking [1], and more well-confined sensations, and by delayed, dull or pressing [1], and less well-confined sensations, respectively.

Selective activation of C-fibres is important considering that chronic pain is associated with C-fibre-mediated activity [2], and being able to induce and evaluate selective activation of C-fibres might have clinical implications for individually tailored management of pain. To be useful, techniques for experimental induction of nociceptive pain in humans should convey non-inflammatory and rapidly transient pain, be reproducible, and preferably also be individually adjustable. Ultra-short laser stimulation with a carbon dioxide (CO_2_) laser meets these criteria and has been used to induce selective activation of C-fibres in humans [3–5], mainly based on different thermal thresholds of Aδ- and C-fibre nociceptors. It has also been used to evoke separate pain responses mediated by Aδ- and C-fibres [3, 5–9], based on differences in nociceptive response latency and neuronal conduction velocity, and assessed by visual analogue scale (VAS) scoring [8, 10], in humans.

This explorative study in humans, based on the use of individually titrated ultra-short laser stimulation enabling separation in time of immediate and delayed pain responses – interpreted, based on their time characteristics, to reflect Aδ- and C-fibre-mediated transmission – was primarily designed for selective induction of delayed nociceptive pain responses, readily assessable by bedside scoring.

## Methods

### Study setting

This prospective preclinical study, approved by the regional Human Research Ethics Review Board (Approval No. LU 337–02, LU 697–03, and LU 2010/160), Lund, Sweden, was carried out in a study setting at Skåne University Hospital, Malmö, Sweden, by three study investigators, one 32-year-old female junior resident physician, and two male senior undergraduate medical students aged 27 and 38 years.

### Subjects

Forty-four (fourteen female) healthy adult volunteers with no current history of pain, reduced sensibility in their lower extremities, or current use of analgesics or other drugs affecting pain perception, were included after normal physical examination. Informed written and verbal consent was obtained from all study participants. The study participants were not allowed to use ethanol or any drug affecting pain perception within 24 h before the study interventions.

### Induction of pain

Nociceptive pain was thermally induced by ultra-short pulsed CO_2_ laser stimulation (10 W effect, 3 mm beam diameter) with a Coherent Ultrapulse 2500C w CPG Laser (Coherent Inc., Santa Clara, California, USA). Each study participant was familiar with the technique of nociceptive stimulation at the time of evaluation, since the laser energy to be consistently used in each participant was determined at least 48 h in advance by incremental five-millisecond increases in pulse duration, starting at 10 ms, until mild intensity levels [11] of immediate and/or delayed pain were consistently induced. Individual levels of laser energy density were calculated from laser effect, pulse duration, and beam diameter. Each participant was subjected to four complete series of nociceptive stimulation by the same study investigator. A series comprised three stimuli, at least one minute apart, located at slightly different skin areas of the plantar arc. The study participants were not informed about their pulse duration, and hence blinded to their individually titrated levels of laser energy density.

### Evaluation of pain

Before individual titration of laser energy density, each participant was carefully instructed how to assess pain intensity levels of immediate (first) and/or delayed (second) pain, following each nociceptive stimulus, on a horizontal 100 mm VAS ruler. Single pain was defined as one reported sensation of mild pain intensity (either immediate or delayed), and double pain defined as two sensations of mild pain intensity, i.e. one immediate and one delayed.

### Statistics

Based on data previously obtained in 35 volunteers evaluating experimentally induced pain by VAS scoring [12], at least 40 study participants had been estimated to be required for analysis of single and double pain responses in the present study.

Parametric data is presented as mean ± standard deviation (SD). Non-parametric data is reported as median with interquartile range (IQR) in parenthesis.

Individual median values with 95% confidence intervals (CI) of immediate and delayed pain intensity during the four series of laser stimuli were calculated from corresponding individual mean values of each series of three. Individual mean values were also tested for order effect with Friedman’s test, and linear regression with a mixed model approach.

The Mann-Whitney U-test was used to compare titrated levels of laser energy density between subjects reporting immediate or delayed pain intensity. The same test was also used to compare female and male subjects with respect to laser energy density and reported pain intensity levels, and in a subanalysis, to compare levels of energy density between subgroups of pain-matched males evaluated by a female or a male investigator.

Levels of probability (*p*) below 0.05 were considered to indicate statistical significance.

## Results

### Subjects

Two female study participants, considered unable to reliably assess the intensity of pain, were excluded. Results were obtained and analysed in the remaining 42 (twelve female) subjects, aged 27 ± 3.4 years and weighing 72 ± 12 kg.

### Induction of pain

There was a tendency towards higher laser energy levels for consistent induction of mild pain in male (median 85 (IQR 60–110) mJ/mm^2^) than in female (median 68 (IQR 51–82) mJ/mm^2^) study participants (*p* = 0.06). Significantly higher titrated levels of energy density (*p* = 0.008) were used in those five males evaluated by a female study investigator (median 224 (IQR 160–253) mJ/mm^2^) than in five males, matched for pain intensity, evaluated by a male investigator (median 74 (IQR 60–81) mJ/mm^2^).

Transient dyschromic spots at the site of dermal stimulation, corresponding to the diameter of the laser beam, disappeared within few days. No other adverse effects were observed or reported.

### Evaluation of pain

The first and second components of double pain responses appeared immediately and at least one second after the laser pulse, and single pain responses were consistently delayed. Twenty-nine (eleven female) study participants consistently reported double pain responses to each nociceptive stimulus, four (one female) reported single pain responses once or twice, and nine (no female) consistently reported single pain responses only.

**Fig. 1:**
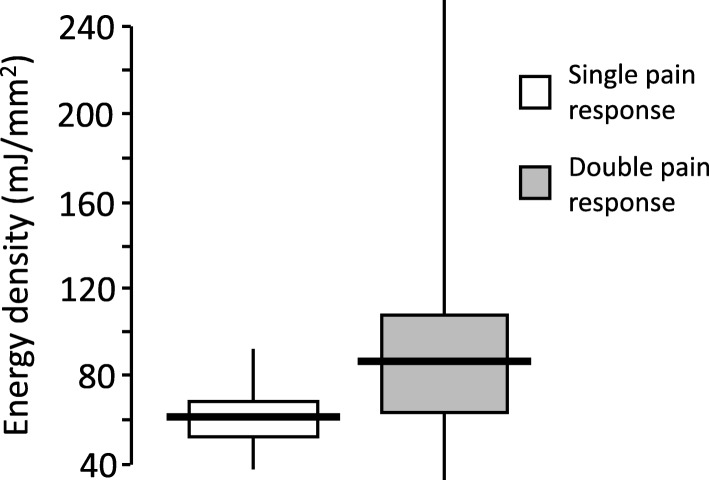
Energy density levels used to induce nociceptive pain by ultra-short CO_2_ laser stimulation in the arc of the foot in 13 (one female) subjects not consistently reporting double pain responses, and in 29 (eleven female) subjects reporting double pain responses of mild intensity. All single pain responses were delayed. Median values are indicated by bold horizontal lines, interquartile range by boxes, and range by vertical lines

Significantly lower (*p* = 0.003) levels of laser energy density were used in subjects with single pain responses than in those with double pain responses (median 61 (IQR 54–71) vs. 88 (64–110) mJ/mm^2^), as shown in Fig. 1.

**Fig. 2:**
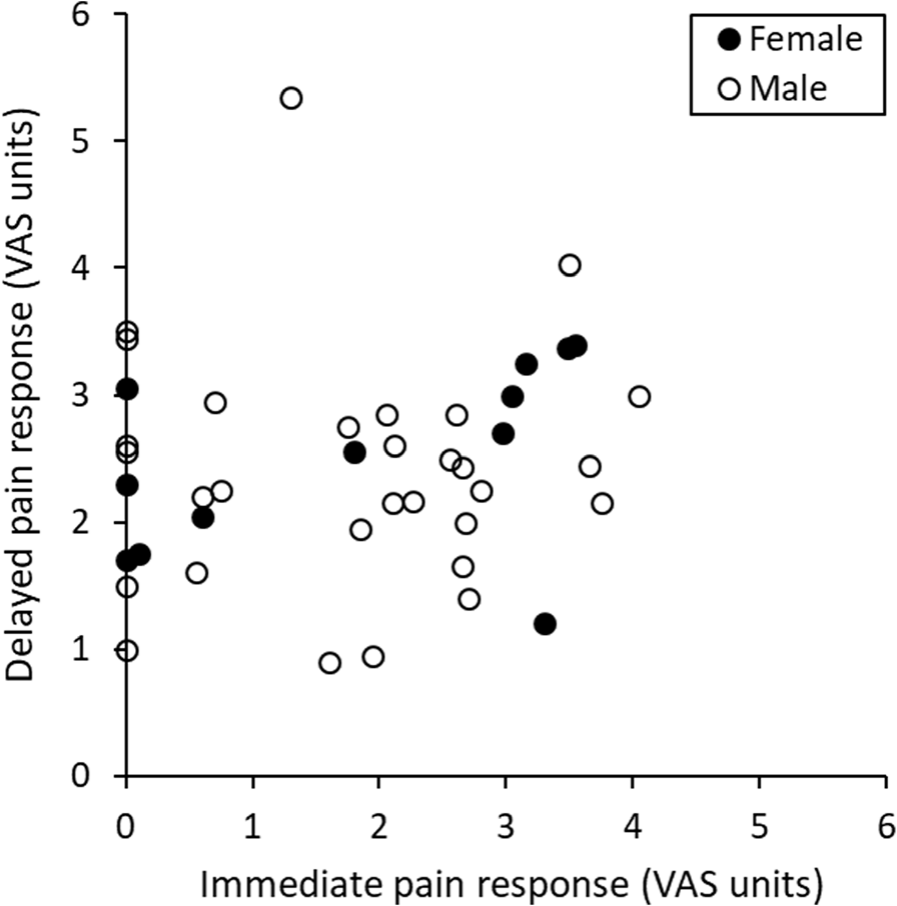
Median intensity levels of immediate and delayed pain induced by ultra-short CO_2_ laser stimulation in the arc of the foot, assessed by individual visual analogue scale (VAS) scoring, in 13 (one female) subjects not consistently reporting double pain responses, and in 29 (eleven female) subjects reporting double pain responses of mild intensity. All single pain responses were delayed

Individually assessed intensity levels of immediate and delayed pain are shown in Fig. 2. There was no difference (*p* > 0.300) in reported levels of pain intensity between female and male study participants (median 2.6 (2.0–3.1) vs. 2.3 (IQR 2.0–2.8) VAS units).

Immediate pain intensity did not differ significantly (*p* > 0.300) between the four series of stimulation (second vs. first − 0.119 (CI -0.374–0.136); third vs. first − 0.144 (− 0.400–0.111); fourth vs. first − 0.245 (− 0.500–0.010)), whereas the intensity of delayed pain was significantly higher (*p* < 0.001) during the first series (second vs. first − 0.445 (− 0.728–-0.163); third vs. first − 0.381 (− 0.663–-0.098), fourth vs. first − 0.567 (− 0.850–-0.285)).

## Discussion

In this study – based on individually titrated ultra-short laser stimulation for induction, and on bedside scoring with VAS for evaluation, of pain – we were able to evoke isolated delayed single pain responses at lower, and separately assessable immediate and delayed pain responses at higher, levels of stimulation intensity.

Influence on peripheral sensitisation and central habituation of pain perception during individual titration, was reduced by gradually increasing the duration of each laser pulse, in agreement with previous recommendations [13, 14]. To avoid epidermal overheating and tissue damage during the study intervention, and to consistently compare pain responses to the same individual level of nociceptive stimulation, we considered it important to use individually titrated, instead of predefined [3, 5, 8, 15, 16], energy levels of laser stimulation, found to induce mild pain intensity, and also to stimulate slightly different skin areas at minute-long intervals.

Thermal induction of pain mediated by Aδ- and C-fibres in humans requires skin temperature levels exceeding activation thresholds of their nociceptors, corresponding to 44.3–46.5 °C versus 41.8–42.4 °C at foot level, i.e. to a difference in heating of two to four degrees centigrade [3]. Those physiological differences at nociceptive receptor level – consistent with our main finding of single pain responses being induced by lower levels of laser energy than double pain responses – enable selective thermal activation of C-fibre nociceptors at temperature levels above their nociceptive threshold and still below that of Aδ-fibre nociceptors [3–5]. Moreover, they most likely explain why all single pain responses in the present study were delayed, i.e. C-fibre mediated.

Our findings of significantly higher laser energy for induction of mild pain in males studied by a female than by a male – enabled by involving more than one study investigator and interpreted to reflect corresponding differences in pain sensitivity – might indicate potential impact of experimenter gender on pain perception. However, this finding in a limited number of study participants was not a predefined aim of this study and should hence be interpreted cautiously. Follow-up studies, preferably with a paired cross-over design, are highly desirable to confirm these findings.

Our approximately one-second difference in time latency between the first and second pain responses strengthens their likelihood of reflecting Aδ- and C-fibre mediated transmission of pain [3, 7, 16], respectively, corresponding to levels of neuronal conduction velocity estimated at 11–19 [3, 15–17] versus 0.7–1.5 [3, 6, 13, 15, 16] m/s. Hence, the short-lasting pulses of laser stimulation used in the present and previous [3, 5–9] studies allow both pain components to be independently evaluated, provided that the temperature thresholds of Aδ- and C-fibre nociceptors are both exceeded. Higher separation in time between the first and second pain responses is achieved by inducing pain in the foot, as done in the present and previous [3, 7, 16] studies, instead of in the hand [1, 4–6, 8–10, 14, 15, 18, 19], considering the up to 35% more delayed transmission of second (C-fibre mediated) pain along the estimated 80–100 cm axonal distance between the plantar arc and the lumbosacral spinal cord [3, 7, 16].

Despite being widely used in research and clinical practice, established bedside tools for pain scoring have been used in few previous studies on nociceptive pain induced by laser stimulation [4, 8, 10, 14, 19], and mainly with assessment of pain intensity on eleven-level visual analogue [10] or numeric rating [4, 14, 19] scales, i.e. with lower resolution than in the present study.

We did not use neuronal or cerebrocortical electrophysiological measurements – in addition to individual pain scoring – to distinguish between first (Aδ-fibre mediated) and second (C-fibre mediated) pain responses, based on differences in their nociceptive response latency and neuronal transmission properties. Nevertheless, the approximately one-second delay of each reported single pain, and second double pain, sensation – interpreted to reflect C-fibre mediated transmission – is consistent with recent neurophysiological findings [10]. Moreover, the induction of two pain responses, consistently well separated in time at bedside, by a single nociceptive stimulus, conforms to simultaneous activation of Aδ- and C-fibre fibres.

Although we did not measure plantar skin temperature – neither before nor after pain stimulation – we do not believe potential individual differences in baseline skin temperature to have considerably influenced our results, since CO_2_ laser stimuli have been shown to induce similar local heating patterns at skin temperatures between 27 °C and 32 °C [3, 14].

Results reported here were obtained in more study participants compared with most previous studies based on laser-induced pain [1, 3–10, 14], except for two previous [16, 18] and one recent [19] studies in similar numbers of subjects. The risk for carry-over effects was reduced by allowing minute-long time intervals between stimulations to avoid overlapping of subsequent pain responses. Furthermore, order effects were diminished and data accuracy improved by using median values of individually calculated average data obtained under identical conditions from repeated series of pain induction. In contrast to earlier similar studies with standardized energy levels (2–4), we used individually titrated levels of laser energy to induce similar and more predictable pain responses and avoid epidermal damage. We consider this readily usable technique of selective C-fibre stimulation and evaluation to be clinically, but not necessarily scientifically, more applicable than spatial filter [20] or dissociating Aδ-fibre nerve block [21] techniques.

## Conclusions

This is the first study to show in humans that individually titrated ultra-short pulses of laser stimulation, enabling separate pain intensity scoring of immediate and delayed responses at bedside, can be used to selectively induce delayed nociceptive pain, most likely reflecting transmission by C-fibres, considering their lower nociceptive thermal threshold, longer nociceptor latency and lower axonal conduction velocity. These findings might promote future research on C-fibre-mediated pain in humans, and possibly also enable more specific evaluation of various analgesic interventions primarily in a research context.

## Abbreviations

CO_2_: Carbon dioxide
IQR: Interquartile range
LU: Lund University
P: Level of statistical probability
SD: Standard deviation
VAS: Visual analogue scale
